# Unveiling the Li/Electrolyte Interface Behavior for Dendrite‐Free All‐Solid‐State Lithium Metal Batteries by *Operando* Nano‐Focus WAXS

**DOI:** 10.1002/advs.202414714

**Published:** 2025-01-31

**Authors:** Yuxin Liang, Fabian A.C. Apfelbeck, Kun Sun, Yingying Yan, Lyuyang Cheng, Guangjiu Pan, Tianle Zheng, Yajun Cheng, Anton Davydok, Christina Krywka, Peter Müller‐Buschbaum

**Affiliations:** ^1^ TUM School of Natural Sciences Department of Physics Chair for Functional Materials Technical University of Munich James‐Franck‐Str. 1 85748 Garching Germany; ^2^ College of Renewable Energy Hohai University Hehai Avenue 1915 Changzhou Jiangsu 213220 P. R. China; ^3^ Helmholtz‐Zentrum Hereon Max‐Planck‐Straße 1 21502 Geesthacht Germany

**Keywords:** all‐solid‐state lithium metal batteries, composite electrolyte, interfacial behavior, *operando* study, x‐ray wide‐angle scattering

## Abstract

Poly(ethylene oxide) (PEO)‐based solid composite electrolytes suffer from poor conductivity and lithium dendrite growth, especially toward the metallic lithium metal anode. In this study, succinonitrile (SN) is incorporated into a PEO composite electrolyte to fabricate an electrode‐compatible electrolyte with good electrochemical performance. The SN‐doped electrolyte successfully inhibits the lithium dendrite growth and facilitates the SEI layer formation, as determined by the *operando* nanofocus wide‐angle X‐ray scattering (nWAXS), meanwhile, stably cycled over 500 h in Li/SN‐PEO/Li cell. Apart from the observation of lithium dendrite, the robust SEI layer formation mechanism in the first cycle is investigated in the SN‐enhanced composite electrolyte by nWAXS. The inorganic electrochemical reaction products, LiF and Li_3_N, are found to initially deposit on the electrolyte side, progressively extending toward the lithium metal anode. This growth process effectively protected the metallic lithium, inhibited electron transfer, and facilitated Li⁺ transport. The study not only demonstrates a high‐performance interfacial‐stable lithium metal battery with composite electrolyte but also introduces a novel strategy for real‐time visualizing dendrite formation and SEI growth directing at the interface area of electrolyte and metallic lithium.

## Graphical abstract

The Li/electrolyte interface behavior is investigated by synchrotron‐radiation based *operando* nano‐focus wide‐angle X‐ray scattering on Li||Li cells facilitating a deeper understanding of dendrite growth and SEI formation. The use of simple additives such as succinonitrile is an effective strategy for enhancing interface stability and improving electrochemical performance in all‐solid‐state lithium metal batteries.

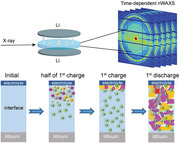

## 1 Introduction

The pursuit of interfacial stable lithium metal batteries (LMBs) has been a longstanding challenge in the field, particularly since metallic lithium replaces graphite as an anode material. Conventional LMBs, which use flammable liquid electrolytes such as ether‐based electrolytes and carbonate‐based electrolytes, pose potential safety hazards like leakage, ignition, and even explosions.^[^ ^1^ ^]^ Furthermore, the highly active lithium metal anode easily reacts with electrolytes, leading to uncontrollable and infinite lithium dendrite growth, which remains the key bottleneck for long‐life stable LMBs.^[^ ^2^ ^]^ The concurrent formation of a solid electrolyte interface (SEI) layer can prevent the direct contact of Li metal and electrolyte. However, the SEI layer can be easily destroyed by its volume expansion, resulting in cell failure. Efforts have been made to address this challenge through various strategies. For instance, researchers have utilized electrolyte solvation chemistry to regulate carbonate‐based electrolytes, achieving stable cycling at low temperatures.^[^ ^3^ ^]^ Additionally, carbonate materials have been used to mediate low‐concentration ether‐based electrolytes, enabling stable cycling at high voltages.^[^ ^4^ ^]^ Furthermore, tuning intermolecular interactions to design nonflammable fluorinated electrolytes for high‐voltage wide temperature cycling^[^ ^5^ ^]^ and building Li‐intercalated interlayers as fast Li^+^ conducting channels and Li protection structure^[^ ^6^ ^]^ can also support the stable high‐voltage cycling of LMBs.

Nevertheless, replacing the organic liquid electrolyte with a solid electrolyte is a crucial step for enhancing safety and expanding the potential applications of LMBs. With all the challenges, significant efforts over the past decades have focused on developing new electrolyte materials, such as sulfide‐based Li_x_PS_y_Cl_6‐y_ argyrodite electrolyte,^[^ ^7^ ^]^ oxide‐based LLZO garnet electrolyte,^[^ ^8^ ^]^ and polymer‐based electrolyte.^[^ ^9^ ^]^ Each type has advantages and limitations. Ceramic electrolytes offer high ionic conductivity but are brittle, while polymer electrolytes are flexible but lack sufficient mechanical strength.^[^ ^10^ ^]^ In contrast, composite electrolytes, consisting of a polymer matrix reinforced with inorganic fillers, inherent the advantages of both polymer and ceramic electrolytes, offering mechanical flexibility along with enhanced stability and conductivity.^[^ ^11^ ^]^ This makes composite electrolytes a promising solution for achieving interfacial stability in lithium metal batteries.

The low‐cost poly(ethylene oxide) (PEO) composite electrolyte stands out as the promising candidate due to its high flexibility, strong Li^+^ solubility, and excellent processability.^[^ ^12^ ^]^ The Li^+^ transport in a PEO‐based electrolyte is realized by the mobility of the ethylene oxide (EO) segments. However, the semi‐crystalline nature of PEO hinders the segment motion dynamics at room temperature, resulting in a low ion conductivity (10^−6^–10^−8^ S cm^−1^).^[^ ^13^ ^]^ To overcome this limitation, introducing low molecular weight plasticizers, such as ester‐based materials and nitrile‐based materials, has been proven as an effective strategy. These plasticizers not only enhance thermal stability and reduce PEO crystallinity, but they also significantly improve ion conductivity.^[^ ^14^, ^15^ ^]^ Furthermore, they contribute to the formation of a stable Li/electrolyte interface composed of LiF, Li₃N, and organic components, which helps to suppress lithium dendrite penetration and improves the overall battery performance.

Currently, adjusting electrolyte compositions has enabled the development of batteries with high‐voltage, fast‐charging, and wide‐temperature capabilities for diverse environmental conditions. However, understanding electrode interfacial behavior, the dynamics of lithium dendrite growth, and the process of SEI formation remains an open challenge despite ongoing research efforts.^[^ ^16^ ^]^ For instance, in‐situ optical microscopy can observe Li‐deposited layer and lithium dendrite under varying current densities,^[^ ^17^ ^]^ while in‐situ transmission electron microscopy has captured the nucleation and growth of Li in functioning solid‐state lithium metal batteries.^[^ ^18^ ^]^ However, these techniques probe only surface information and are limited by the resolution and response times, making it difficult to kinetically capture the dynamical process. Similarly, in‐situ neutron techniques, such as in‐situ neutron tomography,^[^ ^19^ ^]^ face limitations in terms of beam size and beam energy, lacking precision and resolution, preventing the effective monitoring of lithium dendrite and SEI layer formation. In contrast, synchrotron X‐ray techniques offer an innovative approach to studying various types of batteries. For example, Sun et al. used synchrotron X‐ray tomography to capture the degradation of Li‐based anodes during electrochemical cycling.^[^ ^20^ ^]^ They also used this technique to assess the morphological reversibility of modified Li‐based anode materials for next‐generation batteries.^[^ ^21^ ^]^ Similarly, Lu et al. utilized synchrotron X‐ray computed tomography to explore the failure mechanisms of Li/Na–CO_2_ batteries.^[^ ^22^ ^]^ Moreover, nondestructive nanofocus X‐ray scattering (nXS), which combines a high photon flux and a precise illumination area, has been widely applied in fields such as polymer science and solar cells.^[^ ^23^, ^24^ ^]^ The combination of high flux and nm‐grade illumination area provides a unique opportunity to study growth kinetics in real‐time.^[^ ^25^ ^]^ This unique approach gives rise to the visualization of lithium dendrite growth and SEI layer formation process when applied with galvanostatic cycling in LMBs.

In this work, an interfacial‐stable PEO composite electrolyte is fabricated with the addition of succinonitrile (SN) for all‐solid‐state LMBs. The high polarity structure of SN ensures its ability to dissolve various types of salts, which enables an ionic conductivity contribution only from the dissolved salts, as SN itself does not contribute ions in an otherwise non‐ionic matrix. Thus, the addition of SN can inhibit the crystallization of PEO and promote the solubility of lithium salt, resulting in an increase in ion conductivity. With the incorporation of SN, the modified electrolyte demonstrates a high ion conductivity of 2.69 × 10^−4^ S cm^−1^ and a Li^+^ transfer number of 0.57, attributed to the reduced PEO crystallinity of 10.09%. This results in the stable Li plating/stripping at 0.1 mA cm^−2^ over 1000 h and an increased critical current density of 0.7 mA cm^−2^, exhibiting a superior compatibility with metallic lithium. Furthermore, we observe the lithium dendrite growth and the SEI layer formation during the first cycle by *operando* nanofocus wide‐angle X‐ray scattering (nWAXS) using a nanobeam size of 350 nm × 330 nm (H × V). The modified electrolyte in a Li symmetric cell effectively suppresses lithium dendrites by forming a stable interfacial layer primarily composed of LiF and Li₃N. More importantly, the nWAXS reveals that SEI layer formation initiates at the electrolyte side of the Li/electrolyte interface midway through the first cycle and gradually extends toward the lithium metal electrode. Such a stable SEI layer enables steady long‐term charging/discharging at elevated current densities, facilitates ion transports, and mitigates further electrolyte degradation. Leveraging this knowledge, our work presents an easy and cost‐effective strategy for fabricating interfacial‐stable composite electrolytes for all‐solid‐state LMBs and offers insights into the kinetics of electrode/electrolyte interfacial behavior microscopically during cycling.

## 2 Results and Discussion

### 2.1 Ion Conductivity and Thermal Behavior

**Figure 1:**
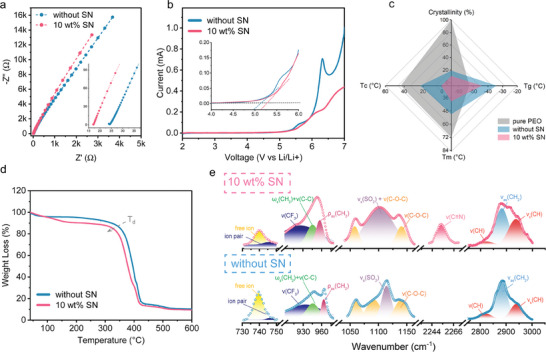
Characterization of ion transport and thermal behavior in PEO‐xSN (x = 0 and 10) composite electrolyte. a) Ionic conductivity of PEO‐xSN at room temperature. b) Electrochemical window of PEO‐xSN electrolyte. c) Summary of glass transition temperature (T_g_), crystallization temperature (T_c_), melting temperature (T_m_), and crystallinity of PEO‐xSN. The pure PEO powder (grey area) acts as the reference for crystallinity. d) TGA ranging from room temperature to 600 °C under Argon atmosphere of PEO‐xSN. e) FTIR spectra of PEO‐xSN film at room temperature.

The influence of SN on the conductivity and electrochemical window is investigated by electrochemical impendence spectroscopy (EIS) and liner sweep voltammetry (LSV), respectively. **Figure** **1**a displays the Nyquist plot of CPEs with and without SN additives. The addition of 10 wt.% SN results in an increased ionic conductivity (2.69 × 10^−4^ S cm^−1^), which is much higher than that of the reference sample without additive (1.22 × 10^−4^ S cm^−1^). As shown in Figure 1b, the introduction of SN expands the electrochemical window to over 5.35 V, thereby reducing the reaction current compared to the reference sample.

To satisfy the operational demands of a lithium battery for commercial applications, the composite electrolyte should exhibit a low glass transition temperature (T_g_) to enhance the conductivity performance, a low melting temperature (T_m_) to guarantee an effective operation at low temperatures, and a low crystallinity to facilitate the ion transport.^[^ ^14^ ^]^ The thermal properties are evaluated using differential scanning calorimetry (DSC) and thermogravimetric analysis (TGA). Pure PEO powder possesses poor thermal properties, as shown in Figure S1 (Supporting Information), which present an extremely high Tm of 72.2 °C and melting enthalpy (ΔH_m_) of 128.06 J g^−1^. The heat flow curves of the reference (PEO‐0SN) sample and PEO‐10SN samples are shown in Figure S2 (Supporting Information). The addition of LiTFSI and Al_2_O_3_ leads to the reduction of T_m_ (51 °C) and ΔH_m_ (50.85 J g^−1^), which can be attributed to the Lewis acid‐base interactions between the ether O of the PEO chain and Lewis acid sites on the surface of Al_2_O_3_,^[^ ^14^ ^]^ resulting to the disruption of PEO crystal structure. A slight increase in T_g_ is observed, attributed to the presence of Al_2_O_3_. Furthermore, the film with SN additive exhibits a further decreased T_g_ of −44 °C, T_m_ of 39.3 °C, and ΔH_m_ of 13.95 J g^−1^. Figure 1c shows the comparison of the samples with/without SN and pure PEO powder as the reference to calculate crystallinity. Notably, the PEO‐10 wt.% SN sample not only presents a lower T_g_, T_m_, and T_c_ but also achieves the lowest relative crystallinity of 10.09%. The decreased crystallinity increases the amorphous portion of PEO. With the addition of SN, the long PEO chains are perturbed, and therefore, the crystalline regions of PEO are disrupted, contributing to the overall ionic conductivity increase by enabling an easier movement of the Li⁺ ions between PEO chains. Figure 1d shows the TGA curve of the composite electrolytes with and without additives. The slight weight loss of 4 wt.% ≈100 °C can be attributed to the removal of moisture or residual solvent in the film. In the reference sample (blue curve in Figure 1d), the major weight loss happens in the temperature window 240 – 520 °C at ≈80 wt.%, indicating sublimation of PEO. In the PEO‐10SN sample, another weight loss of 6 wt.% appears at 100 – 184 °C, which can be attributed to the sublimation process of SN. Notably, not all SN undergoes sublimation. Moreover, the decomposition onset temperature (T_d_) shifts from 363.5 to 347.1 °C after adding SN. This finding can be attributed to the trapping and integration phenomenon of SN in the polymer chain entanglement, and the noncovalent interaction between SN molecules and the functional groups of PEO, respectively.^[^ ^26^ ^]^ Thus, the addition of SN is beneficial for decreasing the melting temperature and crystallinity, contributing to the high conductivity and thermal‐stable composite electrolyte for all‐solid‐state LMBs.^[^ ^27^ ^]^

To further determine the chemical interaction between the PEO matrix and SN, Fourier transform infrared (FTIR) spectroscopy is conducted to analyze the coordination between the functional groups. Figure S3 (Supporting Information) shows the full transmittance FTIR spectroscopy in the range of 450 – 4000 cm^−1^. Figure 1e displays the vibration modes of various groups: (S─N), (─CF_3_), (─CH₂), (─SO_2_), (C─O─C), (C≡N), and (─CH) at wavenumbers 730–750, 900–980, 1030–1200, 2230–2280, and 2750–3050 cm⁻^1^. The characteristic SN peak for the C≡N stretching vibration is clearly observed at 2253 cm^−1^.^[^ ^28^ ^]^ After the addition of SN, the intensity of the S─N vibration from TFSI⁻ at 740 cm⁻^1^ decreases,^[^ ^29^ ^]^ indicating a modification in the Li^+^ solvation structure. This behavior suggests a weakening of the interaction between Li⁺ and PEO, leading to the formation of ion pairs between TFSI⁻ and PEO segments, consequently enhancing the Li⁺ migration ability. The peaks located at 933 cm^−1^ and at 1100 cm^−1^ are corresponding to the ‐CF_3_ rocking mode and to the symmetric ‐SO_3_ stretching mode in TFSI^−^, respectively.^[^ ^28^, ^30^ ^]^ Both peaks exhibit redshifts and altered peak shapes upon SN addition, which indicates enhanced intermolecular interactions between Li⁺ ions and TFSI⁻ anions. The triplet C─O─C stretching modes are observed at 1060, 1090, and 1140 cm^−1^; the peak at 945 cm^−1^ is associated with combined ‐CH_2_ and C─C stretching modes, and the peak at 963 cm^−1^ originates from the mixed asymmetric and symmetric modes of ‐CH_2_.^[^ ^29^, ^31^ ^]^ The ‐CH symmetric and asymmetric peaks are at 2821 and 2939 cm^−1^, while the peak at 2882 cm^−1^ can be attributed to the ‐CH_2_ asymmetric vibration peak.^[^ ^32^ ^]^ With the addition of SN, the mixed ‐CH_2_ bond signal shifts to a lower wavenumber (from 963 to 956 cm^−1^), and the intensity of the symmetric ‐CH_2_ increases. Additionally, the triplet C─O─C peak exhibits a redshift (from 1060 to 1057 cm^−1^) and merges with the ‐SO_3_ stretching peak after the introduction of 10 wt.% SN. These redshifts in the C─O─C peak and ‐CH_2_ peaks indicate that the structure of the PEO chains is modified with the assistance of SN, leading to the reduced crystallinity of PEO and expanded amorphous regions in the PEO matrix.^[^ ^33^ ^]^ This modification enhances the ion conduction and migration, contributing to the improved electrochemical properties of the PEO‐10SN electrolyte for all‐solid‐state LMBs.

### 2.2 Battery Performance

**Figure 2:**
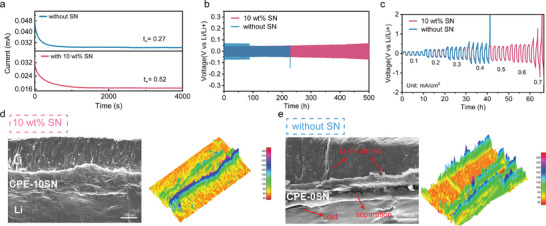
Li symmetric cells performance of PEO‐xSN (x = 0 and 10) composite electrolyte. a) Li^+^ transference number of PEO‐xSN electrolyte. b) First 500 h cycles of Li/PEO‐xSN/Li cells under the current density of 0.1 mA cm^−2^. c) The cycling performance of Li/PEO‐xSN/Li cells under increasing current density. Ex‐situ cross‐section SEM and 3D reconstruction image of d) Li/PEO‐10SN/Li cell and e) Li/PEO‐0SN/Li cell after cycling under 0.1 mA cm^−2^. The color bar in 3D construction images represents the illuminance of this area.

Given its good conductivity and well thermal properties, Li symmetric cells are assembled to evaluate the compatibility of the PEO‐xSN electrolyte with metallic lithium, especially the interfacial stability. The addition of SN improves the electrolyte performance, with the PEO‐10SN electrolyte achieving the highest lithium transference number of 0.52, which is double that of the reference sample (t_⁺_ = 0.27), as shown in **Figure** **2**a. To investigate the long‐term stability, charging/discharging cycling is applied on the Li symmetric cells, with Figure 2b depicting the first 500 h of cycling. Due to the poor ion conducting behavior, the Li/PEO‐0SN/Li cell exhibits a large initial overpotential of 135 mV at 0.1 mA cm^−2^. Then the overpotential suddenly drops to 90 mV at 87 h, followed by a lithium dendrite penetrated short circuit at 220 h, which might be attributed to the micro short circuit inside the cell. In contrast, in the case of the 10 wt.% SN addition, the cell maintains a stable performance over 500 cycles, showing no significant voltage oscillations and sustaining a low overpotential of 80 mV. Additionally, the Li/PEO‐10SN/Li cell shows a superior plating/stripping behavior as the current density increases from 0.1 – 0.7 mA cm^−2^. As shown in Figure 2c, the Li/PEO‐10SN/Li cell exhibits no short‐circuit sign up to 0.7 mA cm^−2^, with a consistently low overpotential at each current density. The symmetric cell with PEO‐0SN shows a short circuit at 0.4 mA cm^−1^, accompanied by a large overpotential of 1160 mV, suggesting a significant increase in the interfacial resistance. This behavior is attributed to the depletion of cations at the interface and the rupture and detachment of the interfacial layer under high current density.^[^ ^34^ ^]^

To further investigate the interface of the Li/electrolyte, scanning electron microscopy (SEM) is conducted on the Li symmetric cells before and after cycling under 0.1 mA cm^−2^. Figure S4 (Supporting Information) shows the cross‐section SEM of two samples before cycling. Three layers can be clearly distinguished as Li metal, composite electrolyte, and Li metal, respectively, and no extra layer can be detected. A distance can be observed between the Li metal and the composite electrolyte, regardless of with or without SN, which means that the contact between the electrolyte and Li metal is not good before cycling. Figure 2d,e, and Figure S5 (Supporting Information) give the SEM images of Li symmetric cells after cycling. The pure Li metal before cycling shows a very smooth surface (Figure S5a, Supporting Information). As shown in Figure 2d and Figure S5b (Supporting Information), after the Li plating/stripping process, the Li metal maintains good contact with the PEO‐10SN electrolyte, and the Li metal exhibits a uniform and flat surface with no visible lithium dendrite formation. This finding suggests a uniform Li plating/stripping in the cycling process. Moreover, the clear high illuminance extra layer can be observed in Figure 2d, denoted as the SEI layer. The 3D reconstruction further confirms the presence of a thick and uniform SEI layer, preventing the dendrite penetration into the electrolyte. In contrast, the symmetric cell without SN addition, seen in Figure 2e and Figure S5 (Supporting Information)c, suffers from dramatic damage during cycling. The lithium metal displays a mossy lithium morphology with voids. The electrolyte shows a clear separation, with large voids at the interface, indicating poor contact with the Li metal. Moreover, two lithium dendrites are observed nucleating from the Li surface, penetrating the electrolyte, and extending into the opposing Li electrode. The 3D reconstruction also reveals the absence of the SEI layer, which might contribute to the failure of the Li symmetric cell with PEO‐0SN electrolyte (without SN additive). These results confirm the crucial role of SN in PEO‐based electrolytes, which increases the Li^+^ migration ability and ion conductivity, thereby contributing to the formation of a stable interface layer and promoting uniform Li^+^ plating/stripping.

The stability of PEO‐10SN with lithium metal is confirmed, and all‐solid‐state LiFePO_4_ batteries are subsequently assembled to assess the oxidative stability of the two samples, as shown in Figure S6 (Supporting Information). The first three cycles are performed at 0.05 C, and the following cycles are performed at 0.1 C. In the Li/PEO‐10SN/LiFePO_4_ cell, the initial 25 cycles can be considered as the activation process. The Li/PEO‐10SN/LiFePO_4_ cell achieves an impressive discharge specific capacity of 169.15 mAh g^−1^ after 25 cycles at 0.1 C, with a coulombic efficiency consistently exceeding 99.5%. Notably, the cell maintains a high capacity retention of 95.6% even after 170 cycles. These results demonstrate that the SN‐modified electrolyte is highly compatible with both lithium metal and LiFePO_4_ electrodes. In contrast, the Li/PEO‐0SN/LiFePO_4_ cell does not exhibit an activation process. It delivers a discharge capacity of 149.56 mAh g^−1^ initially, which decreases to 146 mAh g^−1^ by the 26th cycle. Subsequently, the cell fails during continuous cycling.

### 2.3 Observation of Lithium Dendrite Growth in Li Symmetric Cell

**Figure 3:**
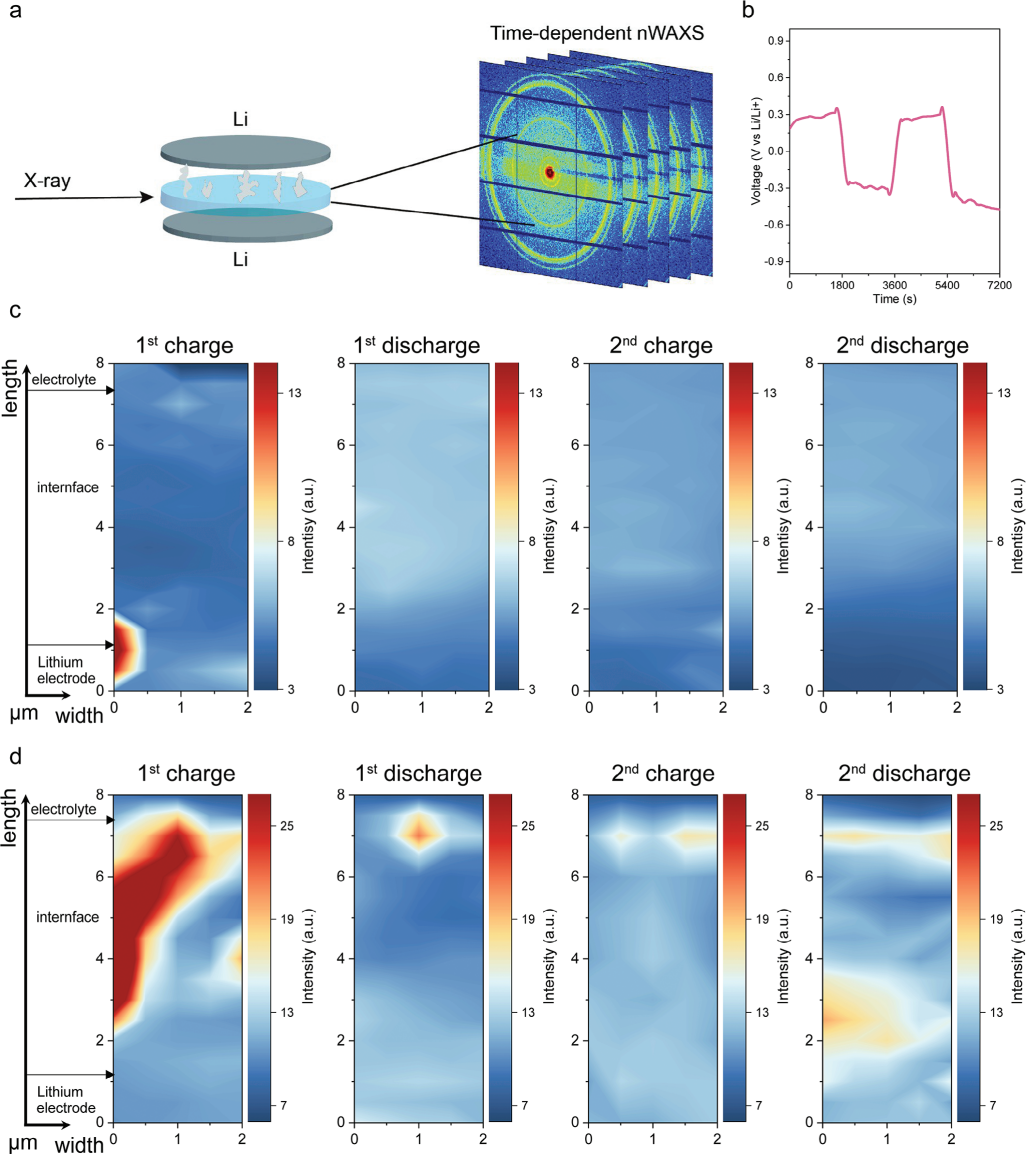
Real‐time observation of lithium dendrites growth on Li/PEO‐xSN/Li cells (x = 0 and 10) during cycling by *operando* nWAXS. a) Schematic illustration of *operando* nWAXS measurement. b) Time‐voltage cycling profile of Li/PEO‐10SN/Li cells during *operando* nWAXS measurement. Temporal 2D contour maps of the Li (110) peak in the scanned area at the Li/electrolyte interface during two charging/discharging cycles of c) Li/PEO‐10SN electrolyte interface and d) Li/PEO‐0SN electrolyte interface. These 2D maps illustrate the growth mechanism of lithium dendrites on the Li/electrode interface.

Lithium dendrites are the most significant hurdles for lithium batteries, especially for those with a metallic lithium anode. While ex‐situ techniques such as SEM or X‐ray tomography can confirm the presence of dendrites after several cycles, capturing the real‐time initiation and growth of dendrites during cell operation remains a difficult task. Nondestructive synchrotron radiation‐based *operando* nWAXS with high photon flux and high temporal resolution,^[^ ^24^ ^]^ stands out from other techniques and enables monitoring the lithium dendrite growth process in real time. Li symmetric cells are cycled at the current density of 0.05 mA cm^−2^. The X‐ray beam is focused on the Li/electrolyte interface, and the scan area is 2 µm × 8  µm (width × length). This area is scanned in 4 × 16 steps to locally resolve structural changes. In combination with a counting time of 20 s, this enables the capture of the spatial evolution during the cycling process (**Figure** **3**a; Figure S7, Supporting Information). The charging/discharging curves of Li/PEO‐0SN/Li and Li/PEO‐10SN/Li cells are shown in Figure 3b and Figure S8 (Supporting Information), respectively. The reference sample exhibits a large overpotential on the initial half cycle, while the symmetric cell with 10wt.% SN additive demonstrates a smooth cycling profile with a lower overpotential. Figure S9 (Supporting Information) gives an example of the nWAXS data treatment. The 2D images obtained from the nWAXS measurements are segmented, as shown in Figure S9a (Supporting Information). The resulting data are then plotted as q (Å^−1^) versus Intensity (a.u.). By analyzing the corresponding peak intensities and positions, temporal contour 2D plots can be generated. Figures S10 and S11 (Supporting Information) show the accumulated 1D radial cuts acquired from the 2D nWAXS data of Li/PEO‐0SN/Li cell and Li/PEO‐10SN/Li cell, respectively, and the standard PDF data of Li, LiF, and Li_3_N.

Figure 3c,d shows the q maps for the prominent Li (110) peak over two charge/discharge cycles of Li/PEO‐10SN/Li and Li/PEO‐0SN/Li cells, respectively, revealing the lithium dendrites formation process. In the Li/PEO‐10SN cell, no visible mossy Li growth is observed along the (110) direction after repeated Li plating and stripping, indicating that dendrites do not form at the Li/PEO‐10SN interface. Moreover, the depletion of Li (200) at the Li/PEO‐10SN electrolyte interface is clearly visible (Figure S12, Supporting Information), indicating that original metallic lithium actively participates in the stripping and plating process from the (200) plane without regrowth. This stable interface in lithium metal batteries guarantees cycling stability, mitigating dendrite growth and promoting uniform lithium deposition. In contrast, in the reference sample (Li/PEO‐0SN/Li cell), large metallic nano Li crystals grow from the metallic lithium side, then distribute along the vertical direction (q_y_ from 2 to 7.5  µm) with respect to the X‐ray beam, and finally reach the electrolyte phase during the first half‐cycle, correlating with the large overpotential seen on Figure S8 (Supporting Information). This leads to the micro‐short circuit within the cells. In the following cycles, the Li (110) remains visible, especially during the second charge and discharge cycles, indicating a continuous growth of the nano lithium crystals at the interface, which ultimately results in cell failure. Unlike the Li/PEO‐10SN/Li cells, where the Li from (200) plane participants in the plating/stripping process, the Li (200) peak from Li/PEO‐0SN interface (Figure S13, Supporting Information) continuously accumulates during cycling, increasing the thickness of the Li layer. This accumulation, in turn, creates an uneven and irregular contact interface, further destabilizing the cell and contributing to its failure.

### 2.4 Observation of Lithium Dendrite Growth in Li Symmetric Cell

A stable and uniform SEI, including components such as LiF, Li_3_N, and Li_2_S, is essential for suppressing lithium dendrite growth, blocking electron transport, and facilitating efficient Li⁺ plating and stripping. However, similar to lithium dendrites, tracking and analyzing the SEI formation process in all‐solid‐state lithium batteries remains challenging. By using *operando* nWAXS, we are able to track the formation kinetics of key SEI components, specifically LiF and Li₃N, in real‐time.

**Figure 4:**
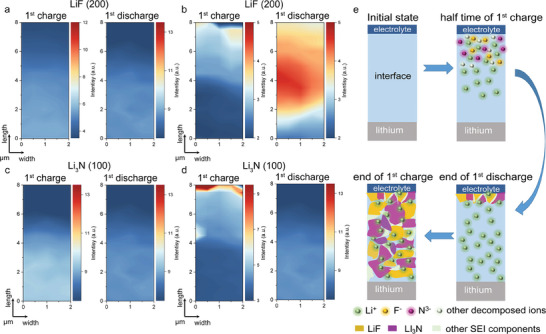
Real‐time observation of SEI formation on Li/PEO‐xSN/Li cells (x = 0 and 10) during the first cycle by *operando* nWAXS. Temporal 2D contour maps of the LiF (200) peaks in the scanned area at the Li/electrolyte interface during the first cycle of a) Li/PEO‐0SN electrolyte interface and b) Li/PEO‐10SN electrolyte interface. Temporal 2D contour maps of the Li_3_N (100) peaks in the scanned area at the Li/electrolyte interface during two charging/discharging cycles of c) Li/PEO‐0SN electrolyte interface and d) Li/PEO‐10SN electrolyte interface. These 2D maps illustrate the growth mechanism of the SEI layer on the Li/electrode interface. e) Schematic illustration of SEI formation mechanism in Li/PEO‐10SN/Li cell. To better illustrate the SEI formation mechanism, the interface area is exaggerated.

The LiF growth on (200) and (111) planes at the Li/PEO‐0SN interface is mapped out in **Figures** **4**a and S14 (Supporting Information), respectively. Interestingly, no signal is detected for the (200) plane, and only a weak (111) plane signal, originating from the metallic lithium side, is observed. The LiF (111) peak might result from the reaction between the decomposition product of TFSI^−^ and Li metal rather than with plating Li^+^. Furthermore, the q map of the (111) plane presents an unclear and irregular edge (Figure S14, Supporting Information), which dissolves during the second half‐cycle (stripping process), indicating the formation of a weak and soft SEI layer. In contrast, at the Li/PEO‐10SN interface, distinct (200) and (111) plane signals are observed (Figure 4b; Figure S15, Supporting Information, respectively). At the Li/PEO‐10SN interface, LiF growth initials from the electrolyte side at around the middle‐time of the Li^+^ plating process, then extends across the interface, reaching the original metallic lithium by the end of the Li^+^ stripping process. This LiF formation leads to the development of a rigid and stable SEI layer, providing enhanced interfacial stability and suppressing dendrite formation. The formation of Li_3_N exhibits a similar trend. At the Li/PEO‐0SN interface (Figure 4c), the signal from the Li_3_N (100) plane is barely detectable. However, at the Li/PEO‐10SN interface, the Li_3_N (100) plane signal is clearly pronounced, as shown in Figure 4d. During the Li^+^ plating process (1^st^ half‐cycle), Li₃N starts forming on the PEO‐10SN electrolyte side and continues to grow toward the metallic lithium electrode. This growth persists throughout the cycle, extending until the Li⁺ stripping process is complete. The formation of LiF and Li_3_N at the Li/PEO‐10SN interface in the 1^st^ charging/discharging process suggests the formation of a stable and continuous SEI layer, which contributes to the high‐performance all‐solid‐state lithium batteries. To wrap up, the SEI formation mechanism is illustrated in Figure 4e. The primary components, LiF and Li_3_N, nucleate from the electrolyte side at the half‐time of the Li^+^ plating process as the result of electrochemical reactions between TFSI^−^ decomposition products and Li^+^. This SEI layer should then gradually extend toward the lithium electrode, continuing its growth until the completion of 1st charge/discharge process. The formation of a stable, ion‐conductive SEI layer is crucial for maintaining the cycling performance and extending the lifespan of lithium batteries.

## 3 Conclusion

In summary, we fabricate a succinonitrile‐enhanced PEO composite electrolyte that effectively improves the conductivity and compatibility toward metallic lithium. We use *operando* nWAXS for real‐time monitoring of the lithium dendrite growth and SEI formation. The addition of SN reduces crystallinity and promotes molecule intercalations, which enhances the ion conductivity. The modified composite electrolyte demonstrates a superior stability against metallic lithium, leading to a low overpotential and stable cycling in all‐solid‐state LMB. *Operando* nWAXS analysis shows that the modified symmetric cells effectively suppress dendrite growth, with no nano lithium crystal detected. In contrast, the reference symmetric cells reveal that the lithium dendrites initially grow in the early stage of the Li^+^ plating process, originating from the lithium metal side and penetrating across the Li/electrolyte interface into the electrolyte. Despite the slight dissolution of nano lithium crystals, these dendrite crystals still exist and remain harmful to the cell performance. Additionally, the SEI formation mechanism is revealed by nWAXS. The main SEI components, LiF and Li_3_N, begin to form from the electrolyte side around halfway through the first charging cycle and continuously extend to the lithium metal side at the end of the first discharging cycle. This process creates a uniform and stable Li/electrolyte interface layer that effectively suppresses the lithium dendrite growth, promoting Li^+^ diffusion and uniform Li^+^ plating/stripping. Thus, our findings validate that the use of simple additives such as SN is an effective strategy for enhancing interface stability and improving electrochemical performance in all‐solid‐state LMBs. Furthermore, the insights gained from *operando* nWAXS measurements will facilitate a deeper understanding of dendrite growth and SEI formation across various battery types, allowing for optimization of interface behavior and electrochemical properties in future research.

## Conflict of Interest

The authors declare no conflict of interest.

## Supporting information



Supporting Information

## Data Availability

The data that support the findings of this study are available from the corresponding author upon reasonable request.
