# Supplementary material for: Unveiling the Li/Electrolyte Interface Behavior for Dendrite‐Free All‐Solid‐State Lithium Metal Batteries by Operando Nano‐Focus WAXS
Source: Adv Sci (Weinh). 2025 Jan 31;12(12):2414714. doi: 10.1002/advs.202414714 (PMC11948068; doi:10.1002/advs.202414714)
Supplement: Supplementary file 1 — Supporting Information [file ADVS-12-2414714-s001.pdf]

ADVANCED SCIENCE
Open Access

Supporting Information

for *Adv. Sci.*, DOI 10.1002/advs.202414714

Unveiling the Li/Electrolyte Interface Behavior for Dendrite-Free All-Solid-State Lithium Metal Batteries by *Operando* Nano-Focus WAXS

*Yuxin Liang, Fabian A.C. Apfelbeck, Kun Sun, Yingying Yan, Lyuyang Cheng, Guangjiu Pan, Tianle Zheng, Yajun Cheng, Anton Davydok, Christina Krywka and Peter Müller-Buschbaum**

Supporting Information

Unveiling the Li/Electrolyte Interface Behavior for Dendrite-Free All-Solid-State Lithium Metal Batteries by Operando Nano-Focus WAXS

Yuxin Liang [1], Fabian A.C. Apfelbeck [1], Kun Sun [1], Yingying Yan [1], Lyuyang Cheng [1], Guangjiu Pan [1], Tianle Zheng [1], Yajun Cheng [2], Anton Davydok [3], Christina Krywka [3], Peter Müller-Buschbaum [1,]*

1 Technical University of Munich, TUM School of Natural Sciences, Department of Physics, Chair for Functional Materials, James-Franck-Str. 1, 85748 Garching, Germany

2 College of Renewable Energy, Hohai University, Hehai Avenue 1915, 213220, Changzhou, Jiangsu Province, P. R. China

3 Helmholtz-Zentrum Hereon, Max-Planck-Straße 1, 21502 Geesthacht, Germany

Corresponding Author

* Peter Müller-Buschbaum – muellerb@ph.tum.de

Experimental Section

Materials

Poly(ethylene oxide) (PEO, average Mv = 600,000 g/mol), Al_2O_3 (< 50 nm nanopowder), lithium bistrifluoromethanesulfonimidate (LiTFSI, 99.95% trace metals basis), succinonitrile (SN, 99%), and acetonitrile (ACN, battery grade, 99.999% trace metals basis) were purchased from Sigma Aldrich. All chemicals were used as received without any other refinement unless otherwise specified.

Preparation of composite electrolyte

The composite electrolyte was fabricated by the solution casting method. PEO and LiTFSI were dissolved in the ACN with a ratio of EO: Li = 14. Afterward, 10 wt% of Al_2O_3 and 10 wt% SN were added, and the abovementioned solution was constantly stirred for 24 h. Then the solution was poured on a Teflon plate, followed by the solution evaporation at the Argon atmosphere for 12 h to obtain a homogeneous free-standing electrolyte.

Cell assembly

CR2032 coin cells were assembled by sandwiching the electrolyte embedded in two stainless steel chips, lithium, and stainless steel electrodes, and two lithium chips for further measurements in an argon-filled glovebox (O_2, H_2O < 0.1 ppm) to avoid any contamination. The assembled cells were heated at 50 °C for 24 h before performing the electrochemical testing.

Characterization

Fourier transform infrared (FTIR) transmittance spectroscopy (Bruker Equinox 55) data was collected at room temperature from 400 to 4000 cm^{-1} (2 cm^{-1} resolution). 256 times spectra were acquired for an acceptable signal–to–noise level. Differential scanning calorimetry (DSC, Mettler Toledo DSC 3) was tested with a scan range of -80 to 100 °C and a heating rate of 10 °C min^{-1}. The degree of crystallinity was obtained by dividing the melting enthalpy of the sample by the enthalpy of the same substance with a crystallinity of a 100%[1], as shown in the following equation:

$$X_c = \frac{\Delta H_m}{\Delta H_{m0}} \times 100\%$$

where ΔH_{m0} refers to the heat enthalpy of pure PEO powder (128.06 J g^{-1}), ΔH_m refers to the heat enthalpy of the reference sample film and PEO-10SN sample film.

Thermogravimetric analysis (TGA, Mettler Toledo TGA/DSC 3+ - MS) was conducted from room temperature to 600 °C, with a ramp of 10 °C min^{-1} under Argon gas. Scanning electron microscopy (SEM, Zeiss SEM EVO15) was performed under an acceleration voltage of 20 kV with a working distance of 10 mm.

The electrochemical measurements of the composite electrolytes were carried out on a VMP300 Biologic electrochemical workstation. For ionic conductivity, the frequency range was from 1 MHz to 0.1 Hz with an AC voltage amplitude of 10 mV and was applied to a symmetric cell with two stainless steel electrodes. The ionic conductivity σ was calculated following a previously reported approach according to the following equation:

$$\sigma = \frac{l}{R\,S}$$

where l refers to the thickness of the electrolyte, R refers to the resistance, and S refers to the electrode area.

The Li^+ transfer number was calculated by the potentiostatic polarization method with 10 mV constant voltage on a lithium symmetric cell according to a previously reported method[2]. The electrochemical impedance spectroscopy (EIS) measurement was performed on the cell before and after polarization. The Li^+ migration number t_{Li+} is then calculated using the following equation:

$$t_{Li^+} = \frac{I_s(\Delta V - I_0 R_0)}{I_0(\Delta V - I_s R_s)}$$

where I_0 and I_s represent the initial and steady-state current after polarization. R_0 and R_s represent the interfacial resistance before and after polarization, and ΔV is the polarization voltage, which in our case is 10 mV.

The linear sweep voltammetry (LSV) test was carried out on the composite electrolytes by placing the electrolyte between a metallic lithium anode and stainless-steel chip and was measured with a sweep rate of 1 mV s^{-1}. The galvanostatic charge/discharge cycling was performed on the NEWARE battery testing system. The Li||Li symmetric cells were cycled under the current density of 0.1 – 0.7 mA cm^{-2}.

***Operando* measurement**

Nanofocus wide-angle X-ray scattering (nWAXS) measurements were performed at the Nanofocus Endstation of beamline P03 (MiNaXS beamline) of the PETRA III storage ring, Deutsches Elektronen-Synchrotron (DESY, Hamburg, Germany)[3] with a nanobeam size of 350 nm × 330 nm (H × V). The beam energy of nWAXS was 12.62 keV. Data was recorded with a Dectris Eigers 9M detector (pixel size 75 µm × 75 µm) located at 216 mm sample-to-detector distance (SDD) to capture the nWAXS signal. For each scanning process, the scan area was 2 µm × 8 µm (16 μm^2), and the scan time was 1h. Each scanning process involved positioning the nanobeam at 4 × 16 points (H × V) across the area, with a step size of 0.5 µm for each step. Four scans were performed, corresponding to two charge/discharge cycles of a

lithium symmetric cell. Each nWAXS scan required one hour to complete, corresponding to the time of half-cycle.

Setup at P03 MiNaXS characterization

The operando setup consisted of a homemade battery cell with two attached Kapton windows on both the front and backside, which was suitable for X-ray transmission measurements at large-scale facilities and an electrochemical characterization with a VMP150 Biologic electrochemical workstation. A Li||Li symmetric cell was used to observe the lithium dendrite growth and SEI layer formation process. Two long screws were used to fix the sandwiched sample and to connect it to the VMP150 Biologic electrochemical workstation. The operando cells were assembled and sealed in an Ar-filled glovebox. Two galvanostatic charge/discharge cycles were applied on each sample with a current density of 0.05 mA cm^{-2}. Figure S5 shows the setup at the Nanofocus Endstation of the P03 beamline.

Data correction and treatment

The nWAXS data were integrated by the DPDAK software[4]. The peak position q and corresponding intensity were extracted for each spot for further analysis. To quantify the distribution of Li, LiF, and Li_3N, the maximum intensity of the individual radial integration line profiles were extracted at q = 2.534 $Å^{-1}$ of Li (110) peak, 3.575 $Å^{-1}$ of Li (200) peak, 3.121 $Å^{-1}$ of LiF (200) peak, 2.703 $Å^{-1}$ of LiF (111) peak, and 2.043 $Å^{-1}$ of Li_3N (100) peak.

Statistical Analysis

The FTIR data were analyzed with Peak Fit software (Systat software, Inc, San Jose, CA, USA) using Gaussian peak-fits. The SEM images were analyzed with Image J software (LOCI, University of Wisconsin, USA), and 3D reconstructions were built with this software. EIS spectra were analyzed with ZView (Scribner Associates, Inc, Charlottesville, VA, USA). The nWAXS data were integrated with the DPDAK software (open source). Results were analyzed with Origin software (Origin Lab, Northampton, MA, USA).

Supporting Figures

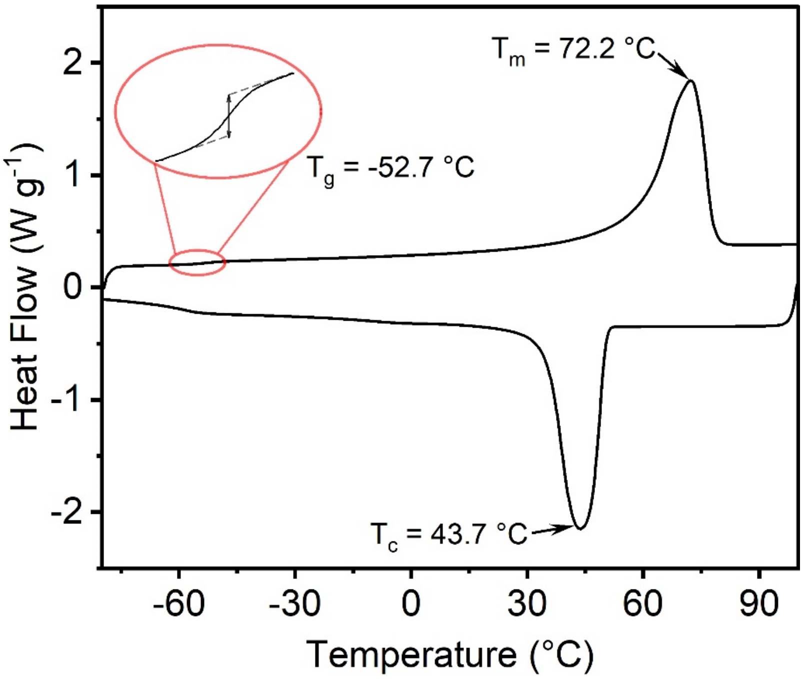

Figure S1. DSC curve of pure PEO powder in the heating/cooling range of -80 to 100 °C. The PEO powder shows a T_g of -52.7 °C, T_m of 72.2 °C, and T_c of 43.7 °C.

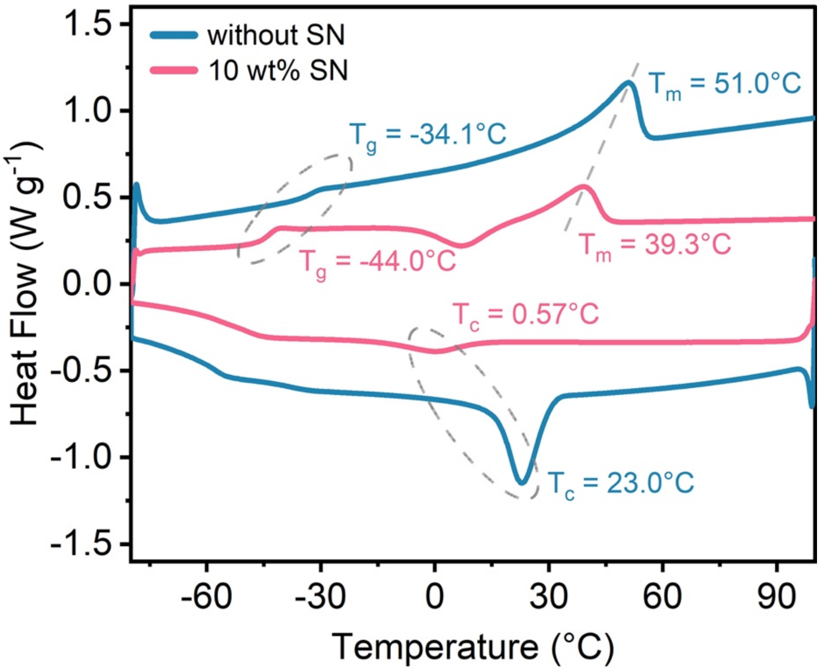

Figure S2. DSC curve of PEO-0SN film (blue curve) and PEO-10SN film (pink curve) in the heating/cooling range of -80 to 100 °C.

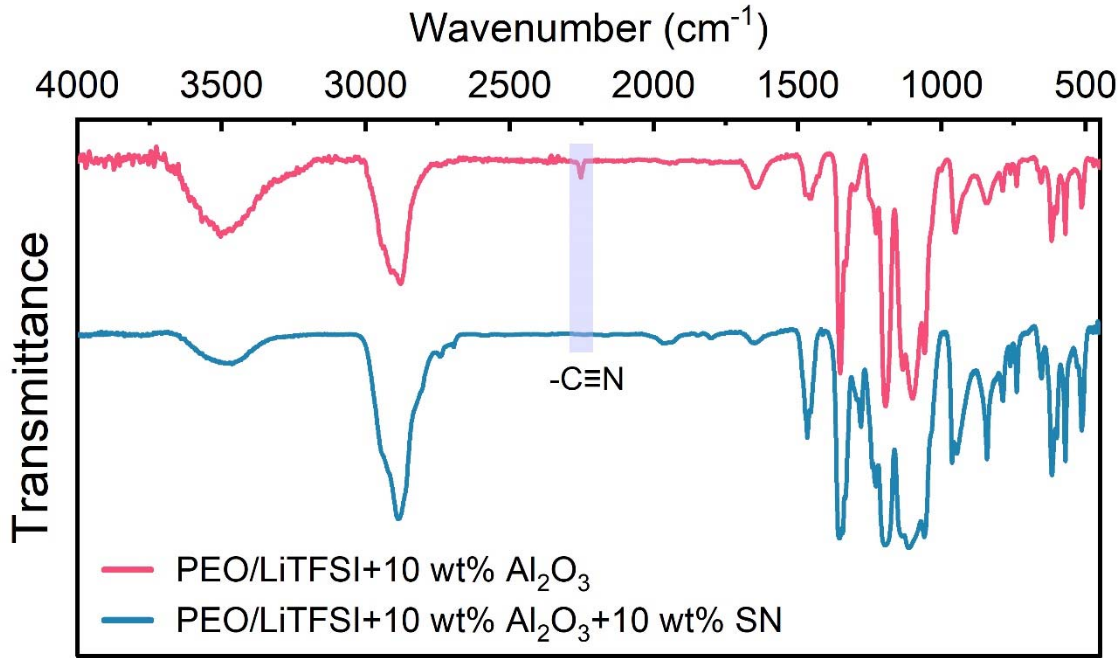

Figure S3. Transmittance FTIR spectra of PEO-0SN film and PEO-10SN film in the range of 400 - 4000 cm^{-1}.

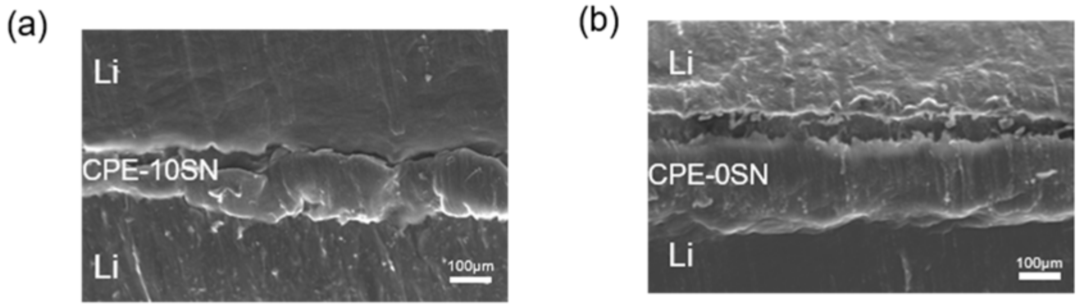

Figure S4. Cross-section SEM image of (a) Li/PEO-10SN/Li cell and (b) Li/PEO-0SN/Li cell before cycling.

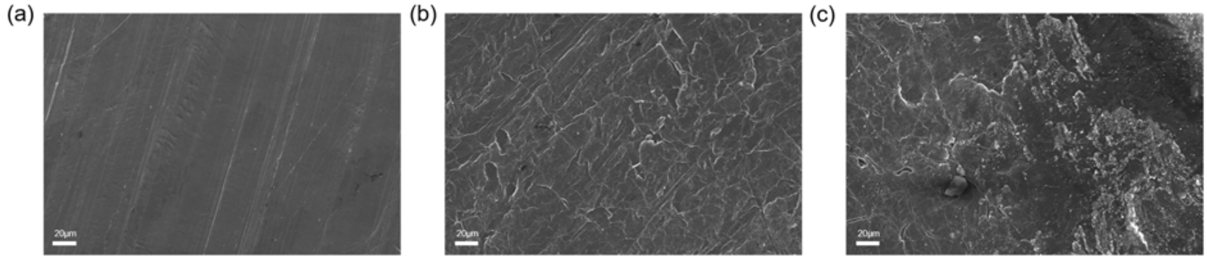

Figure S5. SEM image of Li metal surface: (a) Pure Li metal before cycling, (b) Li metal from Li/PEO-10SN/Li cell after cycling, and (c) Li metal from Li/PEO-0SN/Li cell after cycling.

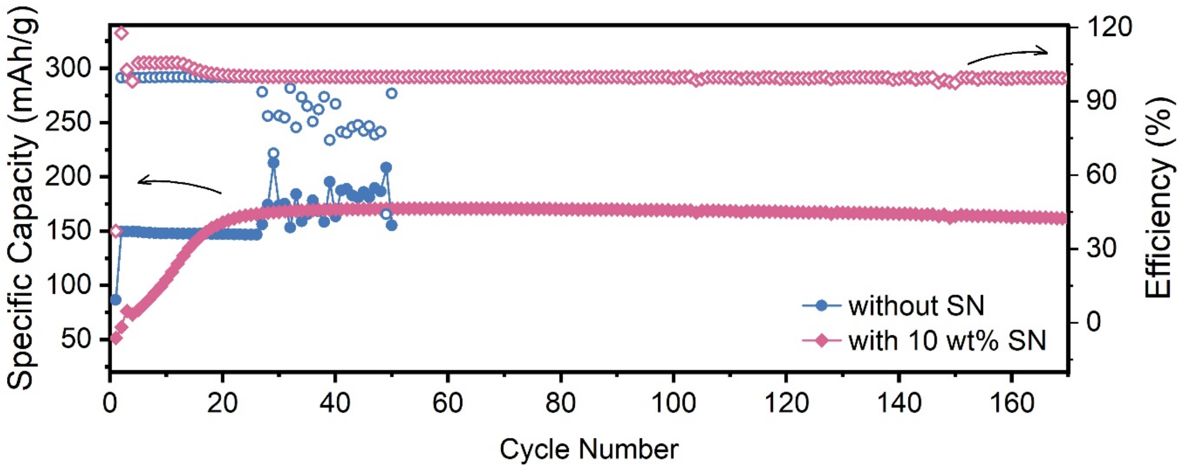

Figure S6. Capacity retention and efficiency of the Li/PEO-10SN/LiFePO4 cell (pink) and Li/PEO-0SN/LiFePO4 cell (blue).

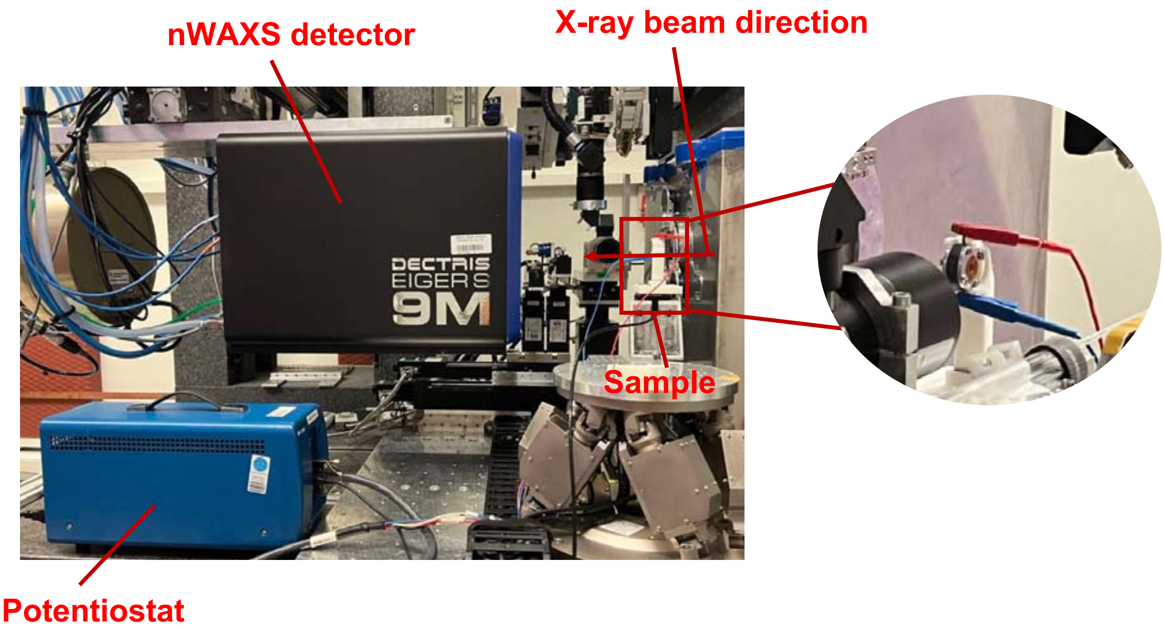

Figure S7. Photo of the set-up used at the Nanofocus Endstation of the beamline P03, at PETRA III synchrotron-radiation source to perform nanofocus wide-angle X-ray scattering. The samples were sealed in homemade cells. The cells were connected with the potentiostat to realize charging/discharging cycling. The X-ray beam transmitted through the Li/electrolyte interface and reached the detector.

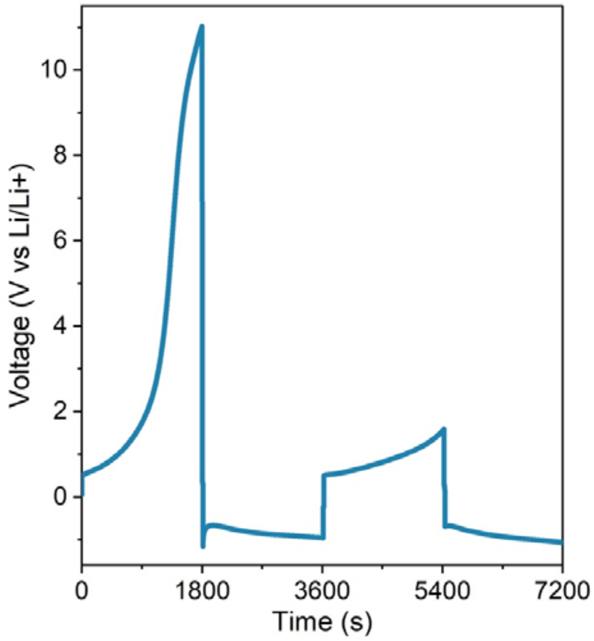

Figure S8. Time-voltage cycling profile of Li/PEO-0SN/Li cells during the *operando* nWAXS measurement.

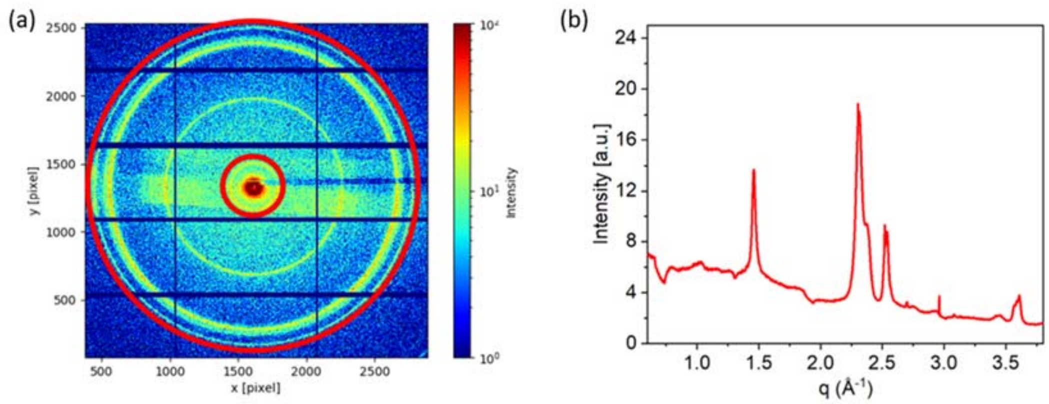

Figure S9. Example of radial cuts in nWAXS data analysis. (a) Exampled 2D nWAXS data, with two red circles framing the area for the integration of the radial cut, and (b) resulting 1D radial cut profile.

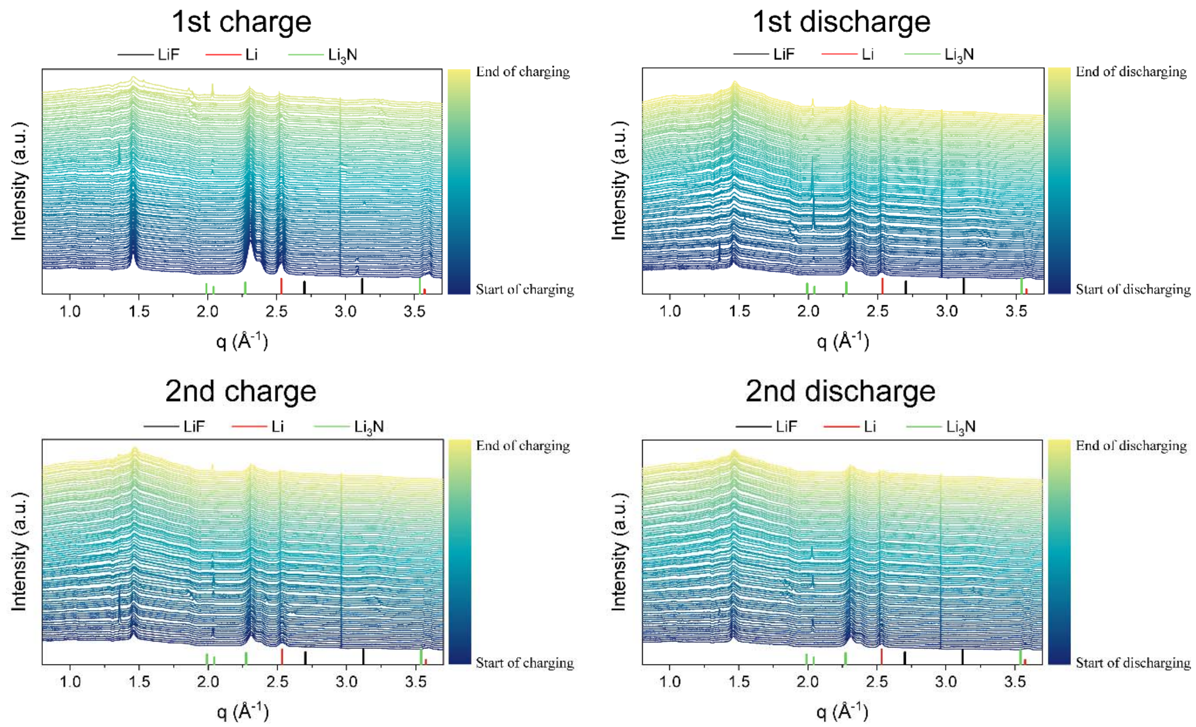

Figure S10. Accumulated radial cuts of 2D nWAXS data at Li/PEO-10SN interface during two charging/discharging cycles. The vertical lines represent the standard pdf profile of Li (black), LiF (red), and Li_3N (green).

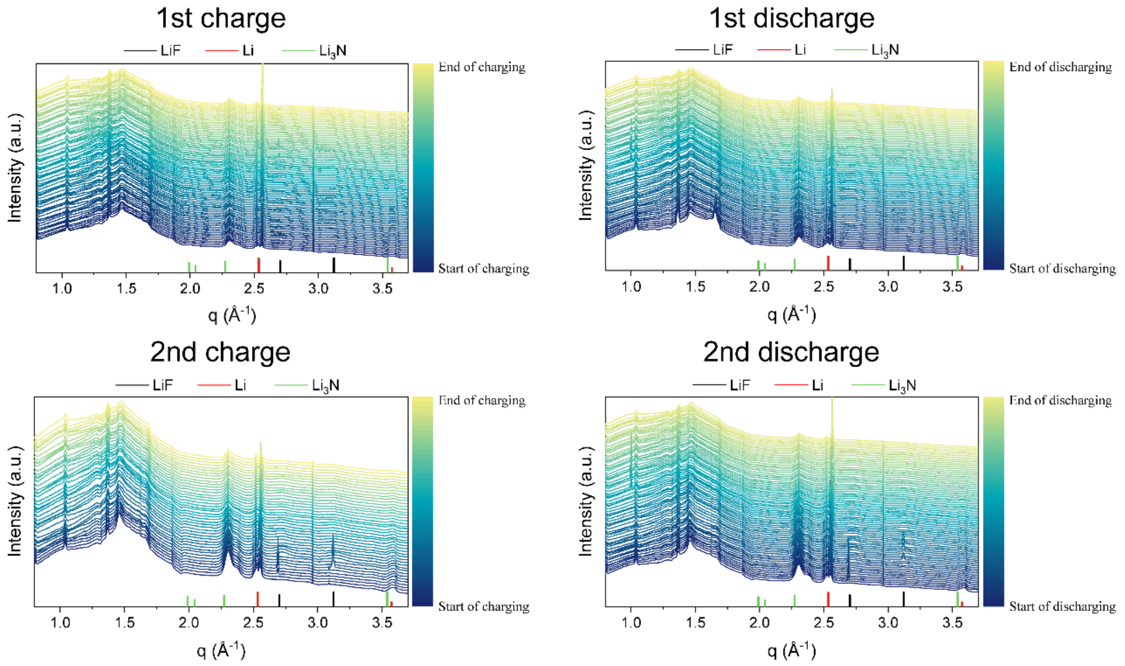

Figure S11. Accumulated radial cuts of 2D nWAXS data at Li/PEO-0SN interface during two charging/discharging cycles. The vertical lines represent the standard pdf profile of Li (black), LiF (red), and Li_3N (green).

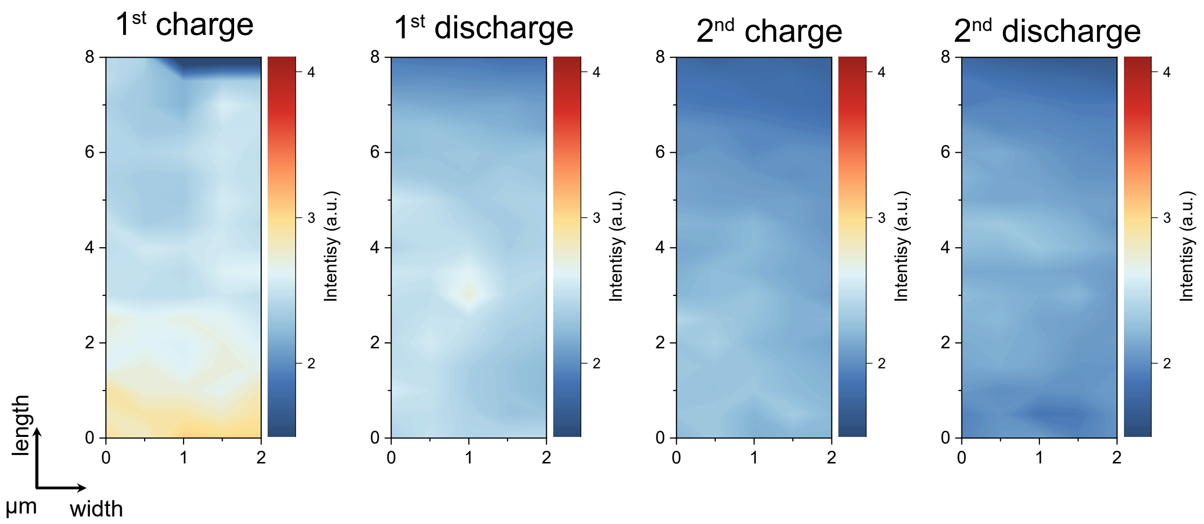

Figure S12. 2D q maps of the Li (200) peak in the scanned area at the Li/PEO-10SN electrolyte interface during two charging/discharging cycles.

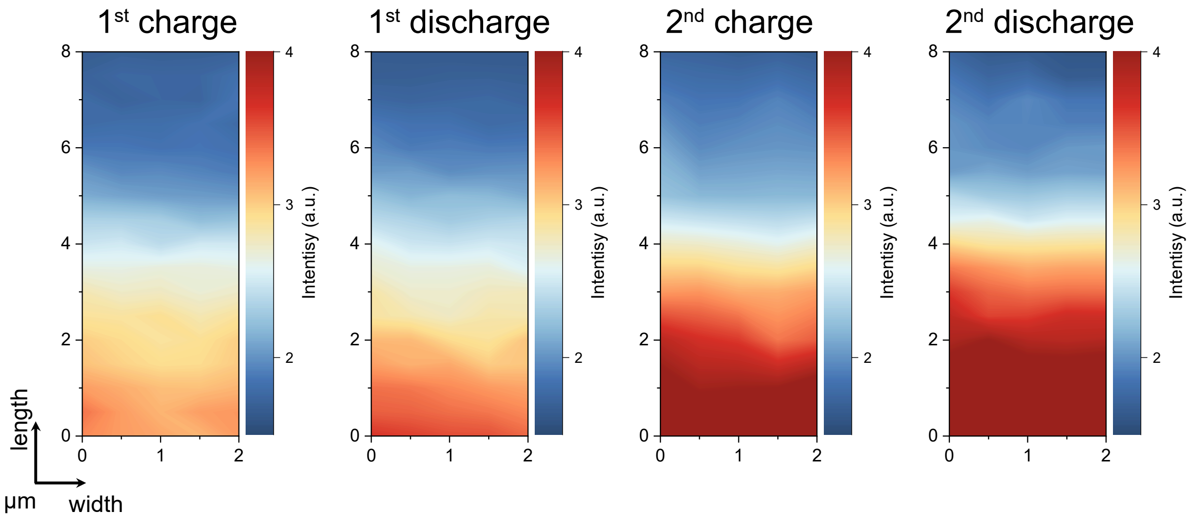

Figure S13. 2D q maps of the Li (200) peak in the scanned area at the Li/PEO-0SN electrolyte interface during two charging/discharging cycles.

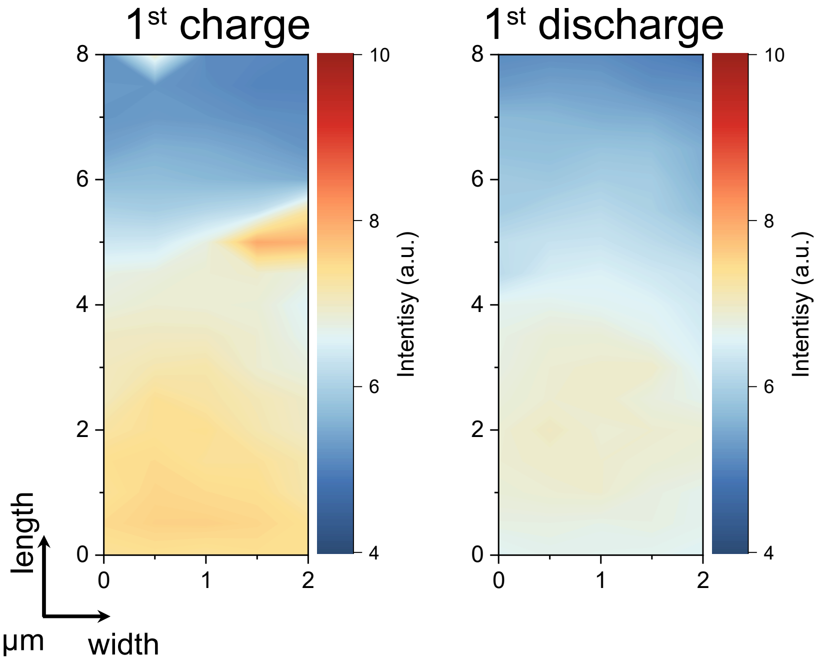

Figure S14. 2D q maps of the LiF (111) peak in the scanned area at the Li/ PEO-0SN interface during the first charging/discharging cycle.

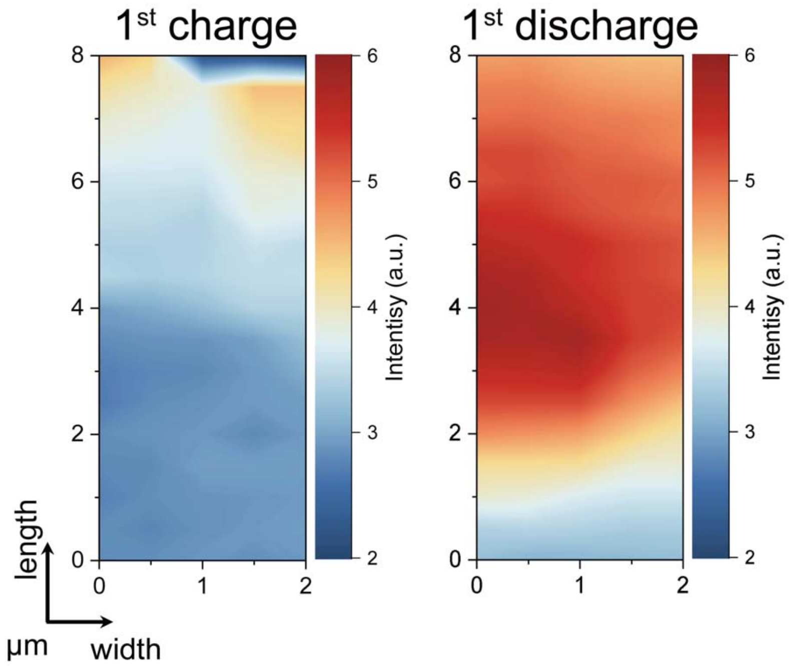

Figure S15. 2D q maps of the LiF (111) peak in the scanned area at the Li/ PEO-10SN interface during the first charging/discharging cycle.

Reference

[1] Y. Kong, J. N. Hay, Polymer 2002, 43, 3873; C. Schick, Analytical and Bioanalytical Chemistry 2009, 395, 1589.

[2] S. Zugmann, M. Fleischmann, M. Amereller, R. M. Gschwind, H. D. Wiemhöfer, H. J. Gores, Electrochimica Acta 2011, 56, 3926.

[3] C. Krywka, J. Keckes, S. Storm, A. Buffet, S. V. Roth, R. Döhrmann, M. Müller, Journal of Physics: Conference Series 2013, 425, 072021.

[4] G. Benecke, W. Wagermaier, C. Li, M. Schwartzkopf, G. Flucke, R. Hoerth, I. Zizak, M. Burghammer, E. Metwalli, P. Müller-Buschbaum, M. Trebbin, S. Förster, O. Paris, S. V. Roth, P. Fratzl, J Appl Crystallogr 2014, 47, 1797.
